# Effects of vitamin D and calcium supplementation on bone mineral density among Thai youth using daily HIV pre‐exposure prophylaxis

**DOI:** 10.1002/jia2.25624

**Published:** 2020-10-11

**Authors:** Krittaporn Pornpaisalsakul, Wipaporn Natalie Songtaweesin, Supatporn Tepmongkol, Prissana Wongharn, Surinda Kawichai, Vichit Suponsilchai, Suvaporn Anugulruengkitt, Thanyawee Puthanakit, the CE‐PID – IHRI Adolescent Study Team

**Affiliations:** ^1^ Department of Paediatrics Faculty of Medicine Chulalongkorn University Bangkok Thailand; ^2^ Center of Excellence for Paediatric Infectious Diseases and Vaccines Faculty of Medicine Chulalongkorn University Bangkok Thailand; ^3^ Department of Radiology Faculty of Medicine Chulalongkorn University Bangkok Thailand; ^4^ Chulalongkorn University Biomedical Imaging Group Faculty of Medicine Chulalongkorn University; ^5^ Division of Endocrinology Department of Paediatrics Faculty of Medicine Chulalongkorn University Bangkok Thailand

**Keywords:** HIV pre‐exposure prophylaxis, young men who have sex with men, young transgender women, bone mineral density, vitamin D, calcium

## Abstract

**Introduction:**

Tenofovir disoproxil fumarate with emtricitabine (TDF/FTC) is used for HIV pre‐exposure prophylaxis (PrEP). TDF may affect bone mineral density (BMD), particularly in youth who are at a stage of peak bone mass accrual. The objective of this study was to evaluate the effect of vitamin D and calcium supplementation on BMD among Thai youth receiving daily oral PrEP.

**Methods:**

This open‐label randomized trial was conducted in male youth aged between 15 and 24 years. Participants were randomized to Arm A who received once‐daily TDF/FTC plus vitamin D3 and calcium supplementation with meals twice daily (400 units of vitamin D3 and 1200 mg of elemental calcium/day) or Arm B who received once‐daily TDF/FTC only. PrEP users were defined as taking at least two tablets/week (tenofovir‐diphosphate level of >350 fmol/punch). Adherence to vitamin D/calcium supplementation was defined as self‐reported adherence of >50%. Lumbar spine (L2‐L4) BMD (LSBMD) was evaluated by dual‐energy X‐ray absorptiometry scan zero and six months after PrEP initiation.

**Results:**

From March 2019 to March 2020, 100 youth were enrolled. Baseline characteristics between the two arms were similar. Median (IQR) age was 18 (17 to 20) years. At entry, median (IQR) LSBMD z‐score was −0.8 (−1.5 to −0.3), 17% had low LSBMD (Z‐score < −2). The median amount of calcium intake from nutritional three‐day recall was 167 (IQR 94 to 272) mg/day, 39% of participants had vitamin D deficiency, defined as 25(OH)D levels <20 IU/mL. At six months, 79 participants were evaluated. Of these, 42 (52%) were PrEP takers and 25 of 38 (66%) of arm A participants had good adherence to vitamin D/calcium supplementation. Significantly higher proportions of youth in arm A compared to arm B had >3% increase in LSBMD at month 6 compared to baseline (67.6% vs. 42.9% respectively; *p* = 0.03). There were significantly higher increases in LSBMD among youth with vitamin D deficiency who were supplemented; arm A + 0.05 (0 to 0.05) compared to arm B + 0.03 (−0.1 to 0.03), *p* = 0.04.

**Conclusions:**

Increases in LSBMD over six months among youth using PrEP who received vitamin D/calcium supplementation was greater than those not supplemented. Long‐term follow‐up should be considered to explore long‐term outcomes.

## 1 INTRODUCTION

Men who have sex with men (MSM) make up the largest population receiving new HIV diagnoses in the United States (87%) [1], a trend also seen in Thailand where this figure is 40% [2], with Thai transgender women (TGW) contributing an additional 12% to national figures [3]. To achieve the ending of AIDS, comprehensive HIV prevention packages and sexual education must be delivered to these key populations [4]. Numerous preventative measures have been used, including the promotion of condom use and reduction of risk behaviours. Medical prevention with oral pre‐exposure prophylaxis (PrEP) has been the latest addition to these strategies.

Tenofovir disoproxil fumarate combined with emtricitabine (TDF/FTC) has been widely used as PrEP [5] in individuals aged 15 years or more [6]. Although generally safe and well‐tolerated, TDF has been associated with a reduction in renal function [7] and bone mineral density (BMD) [8]. Decreases in BMD have been observed in populations living with HIV treated with combination antiretroviral therapy, including TDF‐containing regimens [9], specifically higher tenofovir‐diphosphate (TFV‐DP) concentrations [10]. In addition, several studies have described a small decrease in BMD of uncertain clinical significance among adults using TDF‐based PrEP [10, 11]. The Adolescent Trials Network (ATN) 110 study found similarly modest decreases in BMD in young adults [12]. The Centers for Diseases Control and Prevention (CDC) and World Health Organization (WHO) acknowledge that PrEP use can been associated with a small decrease in bone mineral density (0.5% to 1.5%) in the spine and hip in the first few months of use, which either stabilizes or returns to normal after stopping PrEP use, with the greatest recovery seen among users under 25 years, without evidence of increase in bone fractures seen [13, 14, 15]. The mechanisms of TDF‐related bone toxicity are still unknown, parathyroid hormone (PTH) levels become elevated early after initiation of TDF [16]. Increases in PTH levels are especially seen in those with vitamin D deficiency [16, 17, 18], but also occur in those with adequate vitamin D levels [19]. High plasma TFV‐DP levels are associated with higher levels of vitamin D binding receptors, which may lead to lower free (biologically active) 1,25 hydroxy vitamin D (1,25(OH)D) [17, 20]. Data suggest that TDF induces a state of functional vitamin D deficiency resulting in secondary hyperparathyroidism and increased bone turnover [17, 20]. Bone toxicity is known to be associated with both 25‐OHD insufficiency/deficiency and protective TFV‐DP concentrations, with stronger associations seen in 25(OH)D depletion [18].

In Thailand, PrEP is provided as part of a demonstration project by The Ministry of Public Health of Thailand for key populations, which include MSM and TGW under universal healthcare coverage [21]. It is anticipated this increased accessibility will lead to increased uptake, resulting in clinical safety concerns of PrEP use in youth and young adults that are reaching their stage of peak bone mass accrual [22, 23] a problem compounded by already high rates of vitamin D deficiency in the Thai population [24, 25]. There is currently a dearth of information on the impact of PrEP use on growth in youth and a concern for both users and prescribers [8].

It is known that vitamin D supplementation improves TDF‐associated PTH elevations [19] and vitamin D plus calcium supplementation improves endocrinological and BMD changes associated with TDF use as treatment in HIV infection populations [26, 27, 28]. To date, there are limited data demonstrating the effect of vitamin D and calcium supplementation in youth PrEP users. This study aimed to evaluate the effects of vitamin D and calcium supplementation on BMD among Thai youth receiving daily oral PrEP.

## 2 METHODS

### 2.1 Study design

This study was a randomized, open‐label clinical trial conducted in youth aged 15 to 24 years at the King Chulalongkorn Memorial Hospital, Bangkok, Thailand. The study protocol was approved by the Institutional Review Board of Chulalongkorn University (IRB Number 740/61). Informed consent was obtained from participants before enrolment with a waiver of parental consent for those aged <18 years. The study was registered at the Thai Clinical Trials Registry (TCTR), study number TCTR20190624002.

### 2.2 Participants

Young MSM (YMSM) and young transgender women (YTGW) aged 15 to 24 years presenting for voluntary HIV testing at the Thai Red Cross Anonymous Clinic or King Chulalongkorn Memorial Hospital (KCMH) were recruited. Inclusion criteria included being PrEP‐naïve, seronegative HIV status, having HIV risk acquisition attributes, including: multiple sex partners, inconsistent condom use or HIV seropositive partners; willingness to initiate PrEP, male sex assignment at birth and self‐identification as MSM or TGW. Exclusion criteria included: participants with symptoms of acute retroviral syndrome, history of bone fracture, history of receiving calcium (more than 1000 mg of elemental calcium/day) and/or vitamin D (more than 400 IU/day) supplementation within the preceding six months, growth hormone deficiency, endocrinopathies including hyperparathyroidism, hypoparathyroidism, Cushing syndrome, creatinine clearance <60 mL/min/1.73 m^2^ or a history of systemic steroid use.

### 2.3 Study procedures

Study participants were allocated by blocks of four randomization with the concealment method using an opaque sealed envelope technique, at a ratio of 1:1 into two groups. Arm A received once‐daily TDF/FTC (300/200 mg) plus vitamin D and calcium supplementation with meals twice daily (Oskept^®^; 600 mg of elemental calcium and 200 units of cholecalciferol), Arm B received once‐daily TDF/FTC (300/200 mg) only.

Lumbar spine (L2‐L4) BMD (LSBMD) measurements were performed within one month of PrEP initiation and at month 6. Laboratory studies, questionnaires to evaluate physical activity, sun exposure and calcium intake were performed at enrolment and month 6. Physical activity questionnaires (PAQ) were based on Kowalski *et al*. [29] and calculated a score between 1 and 5, 1 indicating low, >1 to <5 moderate and 5 high physical activity.

Sun exposure questionnaires asked about how much time participants spent outdoors, sunscreen use between 10 am to 4 pm each week and from this [30], calculated an average daily figure. Average calcium and energy intake were evaluated using three‐day food recall questionnaires that participants were asked to complete prior to clinic visits using the software INMUCAL‐Nutrients V4.0.

Drug adherence evaluation was done at months 3 and 6 using venous tenofovir‐diphosphate (TFV‐DP) levels, TFV‐DP levels of >350 fmol/punch were taken to represent the use of at least two tablets of PrEP/week [31]. Adherence to calcium/vitamin D supplementation was defined as self‐reported adherence of >50% on average over the six‐month follow‐up period [32].

### 2.4 Bone mineral density assessment and interpretation

LSBMD measurements were performed using dual‐energy X‐ray absorptiometry (DXA) scanners, Hologic Discovery A Bone Densitometer (Hologic Inc, Bedford, MA, USA), or Hologic Horizon W Bone Densitometer (Hologic Inc, Bedford, MA, USA) at King Chulalongkorn Memorial Hospital. These machines were calibrated daily according to manufacturer’s instructions. Cross‐calibration between these two machines was conducted prior to use to confirm figures gained from them were directly comparable [33]. BMD measurements were reported in g/cm^2^. BMD Z‐scores were calculated from age‐ and sex‐matched reference BMD values for Caucasian children provided by the machine manufacturer [34]. Low BMD was defined as BMD Z‐scores −≤2. The proportion of cases with <3% increase of BMD from baseline at six months was determined, as the annual rates of change for late‐stage adolescents is +3% for BMD [35].

### 2.5 Laboratory measurements

Serum 25(OH)D and iPTH levels were measured by chemiluminescent microparticle immunoassay. A 25(OH)D level <20 ng/mL and 20 to 30 ng/mL were defined as vitamin D deficiency and insufficiency respectively [24]. The upper normal limit of serum iPTH used was 65 pg/mL [36]. Serum for 25(OH)D and iPTH stored at −70°C until analysis. Serum calcium, phosphorus and alkaline phosphatase were measured using the VITROS Ca, VITROS PHOS and VITROS ALKP slide methods respectively.

TFV‐DP concentrations were measured using liquid chromatography mass spectrometry (LC‐MS/MS). Venous blood was collected in EDTA‐K2 tubes and 25 µL was spotted onto Whatman Protein Saver 903 cards (minimum 3 spots/card) and dried for at least three hours. Dried blood spots were stored at −70°C until analysis. For TFV‐DP quantification, a 3mm punch was taken from a single spot using a micro‐puncher. The dried blood was lysed from the paper using methanol: H2O and then extracted and purified using Solid Phase Extraction (SPE). TFV‐DP was eluted through the SPE cartridge and then dephosphorylated using alkaline phosphatase. The resulting tenofovir (TFV) was then purified and eluted via SPE and the samples analysed using LC‐MS/MS. The TFV‐DP calibration curve range was 200 to 10 000 fmol/3 mm punch [37].

### 2.6 Statistical analysis

The sample size of this study was calculated based on the assumption that differences in percent change in LSBMD between two groups were 70% [11], with 90% power of test, alpha 0.05 and a ratio of participants of 1:1 using the two‐sample means test equation, which indicated that at least 88 participants were needed for the study, 44 for each arm. When a 10% rate of loss to follow‐up was accounted for, it was calculated that a total of 100 participants were needed for the study.

Continuous variables data were reported as medians (interquartile ranges [IQRs]) and categorical variables as numbers and percentages. Comparison of LSBMD, LSBMD Z‐scores, 25(OH)D, iPTH and calcium levels between baseline and six months were done using the Wilcoxon signed‐rank test.

The Wilcoxon rank sum test was used to compare arms A and B. Results were considered statistically significant when *p* < 0.05. Analyses were conducted using STATA software version 13.

## 3 RESULTS

From March 2019 to March 2020, 100 youth who initiated PrEP were enrolled, 66 were YMSM (66%) and 34 YTGW (34%). Baseline characteristics are shown in Table 1. Median (IQR) age was 18 (17 to 20) years at enrolment, median (IQR) BMI was 20.5 (18.7 to 22.4) kg/m^2^. Twenty‐eight percent of participants reported some sun exposure during 10 am to 4 pm. Most had moderate physical activity and median calcium intake was 167 (94 to 272) mg/day. Fourteen (41%) of TGW used feminizing hormone therapy (FHT), which may have led to lower plasma TFV‐DP concentrations [38].

**Table 1:** Baseline characteristics, bone mineral density and bone markers of youth initiating daily tenofovir/emtricitabine as daily HIV preexposure prophylaxis

Characteristics	Total (n = 100)	TDF/FTC + Oskept^®^ (n = 50)	TDF/FTC (n = 50)	*p*‐value^a^
Sexual orientation				
MSM; n (%)	66 (66)	34 (68)	32 (64)	0.67
TGW; n (%)	34 (34)	16 (32)	18 (36)	
Age, years	18 (17 to 20)	18 (17 to 19)	18 (17 to 21)	0.55
BMI, kg/m^2^	20.5 (18.7 to 22.4)	20.4 (18.7 to 22.4)	20.5 (18.7 to 22.2)	0.94
Sun exposure (10 am to 4 pm)				
Sun exposure; n (%)	28 (28)	14 (28)	14 (28)	0.99
Hour(s)/day; median (IQR)	0.5 (0.2 to 0.9)	0.4 (0.2 to 0.7)	0.6 (0.4 to 0.9)	0.23
Physical activity level				
Low activity; n (%)	21 (21)	13 (26)	8 (16)	0.22
Moderate activity; n (%)	79 (79)	37 (74)	42 (84)	
Daily calcium intake, mg/day:	167 (94 to 272)	167 (95 to 298)	168 (93 to 266)	0.55
Bone mineral density^b^				
LSBMD, g/cm^2^; median (IQR)	0.93 (0.84 to 1.00)	0.94 (0.82 to 1.00)	0.93 (0.85 to 1.00)	0.94
LSBMD z‐score; median (IQR)	−0.8 (−1.5 to − 0.3)	−0.8 (−1.5 to − 0.3)	−0.8 (−1.8 to − 0.3)	0.52
LSBMD z‐score ≤−2; n (%)	17 (17)	12 (24)	5 (10)	0.06
Alkaline phosphatase, U/L	75.0 (61.0 to 87.5)	68.5 (59.0 to 86.0)	79.0 (66.0 to 93.0)	0.15
Calcium, mg/dL	9.9 (9.5 to 10.1)	9.9 (9.6 to 10.1)	9.9 (9.5 to 10.0)	0.49
Phosphorus, mg/dL	3.5 (3.1 to 3.9)	3.4 (3.1 to 3.9)	3.5 (3.0 to 3.9)	0.68
25 OHD, ng/mL	21.4 (17.3 to 25.9)	21.4 (18.4 to 26.0)	21.9 (17.1 to 25.7)	0.81
25 OHD < 20 ng/mL	39 (39)	18 (36)	21 (42)	0.54
iPTH, pg/mL	39.5 (30.5 to 52.6)	38.7 (30.3 to 50.2)	41.3 (30.8 to 57.8)	0.76
iPTH > 65, pg/mL	14 (14)	6 (12)	8 (16)	0.56

Data shown as medians (Interquartile ranges) or number (%). 25 OHD, 25 hydroxyvitamin D; BMI, body mass index; iPTH, intact parathyroid hormone; LSBMD, lumbar spine bone mineral density; MSM, men who have sex with men; TGW, transgender women.

^a^ *P* value calculated by Wilcoxon signed‐rank sum test

^b^ total n = 99; arm A n = 49, arm B n = 50.

At baseline, the median (IQR) LSBMD z‐score was −0.8 (−1.5 to −0.3), and 17% had low LSBMD z‐scores (LSBMD z‐score ≤ −2) (Table 1). Overall, baseline serum 25(OH)D concentration was 21 (17 to 26) ng/mL and 39% of participants had vitamin D deficiency. Fourteen percent of participants had high iPTH levels (five participants with vitamin D deficiency and nine with vitamin D insufficiency).

Seventy‐nine subjects (79%) completed six months of study follow‐up, 13 participants in arm A and eight participants in arm B were lost to follow‐up due to relocation during the study with a median follow‐up duration of four months (IQR 3 to 5).

### 3.1 PrEP and vitamin D/calcium intake during study

At month 3, overall median (IQR) TFV‐DP concentration was 646 (200 to 1572) fmol/punch, arm A was 337 (200 to 1572) fmol/punch and arm B was 835 (207 to 1588) fmol/punch (*p* = 0.19). There were 44 (57%) participants with TFV‐DP concentrations >350 fmol/punch, consistent with the use of two to three tablets of TDF/FTC per week; 20 (49%) in arm A and 24 (67%) in arm B (*p* = 0.11).

At month 6, overall median (IQR) TFV‐DP concentration was 371 (200 to 1458) fmol/punch, arm A was 307 (200 to 1327) fmol/punch and arm B was 822 (200 to 1514) fmol/punch (*p* = 0.50). Of these, 42 (52%) of participants had TFV‐DP concentrations >350 fmol/punch; 17 (44%) in arm A and 25 (60%) in arm B (*p* = 0.15).

In arm A, 66% of participants self‐reported adherence at month 6 to vitamin D and calcium supplementation >50% of the time. Despite imperfect PrEP adherence, there were no HIV seroconversions in this study.

### 3.2 BMD Z‐score and BMD changes

**Figure 1:**
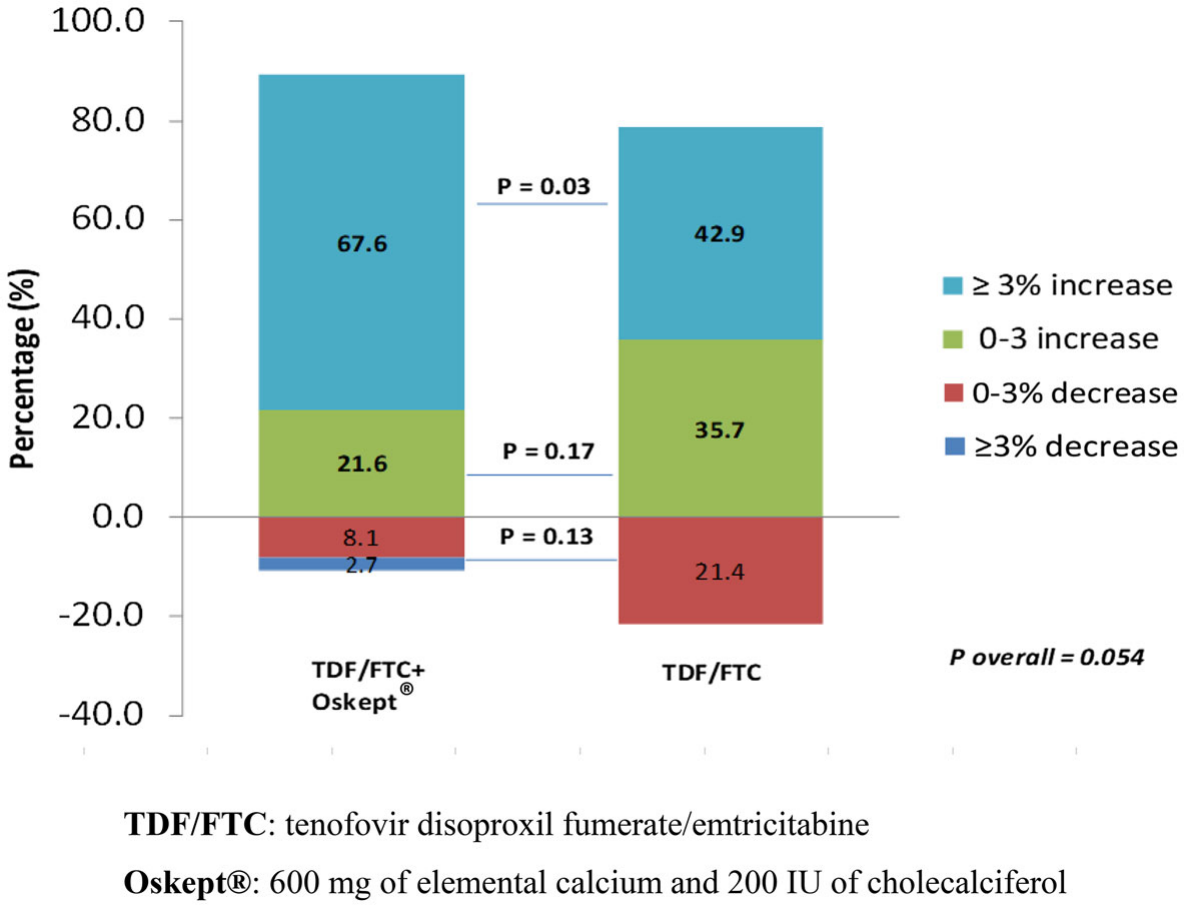
Change of bone mineral density over six‐month period among youth taking oral daily HIV preexposure prophylaxis. *p*‐value comparison using the chi‐squared test. TDF/FTC, tenofovir disoproxil fumerate/emtricitabine; Oskept^®^, 600 mg of elemental calcium and 200 IU of cholecalciferol.

The majority of participants in both groups had increased LSBMD over the six‐month follow‐up period (Figure 1). Approximately two‐thirds (67.6%) of the supplemented group saw a ≥3% increase in their LSBMD in six months, also seen, albeit to a lesser extent, in the non‐supplemented group (42.9%). Decreased in LSBMD was seen in 10.8% and 21.4% of the supplemented and non‐supplemented groups respectively (*p* = 0.054). LSBMD within arms increased between baseline to month 6; with a median change in LSBMD + 0.04 (IQR 0.02 to 0.05 *p* < 0.001) in arm A and +0.02 (IQR 0.01 to 0.03, *p* < 0.001) in arm B. The median change in LSBMD was higher in arm A with Oskept^®^ supplementation, +0.04 (IQR 0.02 to 0.05) compared to arm B +0.02 (IQR 0.01 to 0.03) (*p* = 0.02) (Table 2).

**Table 2:** Comparison of bone mineral density and biochemical makers by randomization arm

Parameters	TDF/FTC once daily + Oskept^®^ twice daily (N = 37)			TDF/FTC once daily (N = 42)			*p*‐value**
	Median (IQR)	Change Median (IQR)	*p*‐value*	Median (IQR)	Median change (IQR)	*p*‐value*	
LSBMD (g/cm^2^)							
Month 0	0.92 (0.82 to 1.00)	0.04 (0.02 to 0.05)	<0.001	0.95 (0.85 to 1.01)	0.02 (0.01 to 0.03)	<0.001	0.02
Month 6	0.97 (0.84 to 1.03)			0.98 (0.86 to 1.05)			
LSBMD z‐score							
Month 0	−0.9 (−1.7 to − 0.4)	0.3 (0 to 0.4)	<0.001	0.6 (−1.5 to − 0.1)	0.1 (−0.1 to 0.3)	0.007	0.25
Month 6	0.6 (−1.4 to − 0.2)			0.6 (−1.4 to 0.2)			
25 OHD, ng/mL							
Month 0	21.3 (18.9 to 26.0)	0.1 (−4.9 to 3.6)	0.92	21.2 (17.2 to 24.9)	−1.7 (−3.1 to 1.5)	0.08	0.43
Month 6	21.5 (18.3 to 25.8)			20.4 (16.0 to 24.4)			
iPTH, pg/mL							
Month 0	38.7 (31.3 to 53.5)	4.9 (−4.2 to 13.5)	0.08	40.8 (30.8 to 57.8)	5.8 (−6.7 to 20.8)	0.07	0.77
Month 6	44.1 (33.1 to 64.6)			45.0 (35.4 to 62.6)			
Calcium, mg/dL							
Month 0	9.9 (9.6 to 10.1)	−0.1 (−0.4 to 0.1)	0.11	9.9 (9.5 to 10.0)	−0.2 (−0.5 to 0.1)	0.11	0.93
Month 6	9.6 (9.4 to 10.0)			9.7 (9.4 to 9.9)			
Phosphorus, mg/dL							
Month 6	3.3 (3.1 to 3.9)	0 (−0.3 to 0.4)	0.57	3.5 (3.0 to 3.7)	0.3 (0 to 0.7)	0.001	0.06
Month 6	3.5 (3.2 to 3.8)			3.7 (3.4 to 3.9)			
ALP, U/L							
Month 0	69 (59 to 87)	−0.5 (−8 to 7)	0.94	79 (66 to 87)	−1 (−7 to 8)	0.62	0.68
Month 6	71 (57 to 88)			76 (65 to 86)			

25 OHD, 25 hydroxyvitamin D; intact parathyroid hormone; iPTH; LSBMD; lumbar spine bone mineral density.

* *p*‐value comparison between months 0 and 6 within arms done with the Wilcoxon signed‐rank test.

** *p*‐value for comparing median difference between arms done with the Wilcoxon rank‐sum test.

In order to explore effect of PrEP on BMD, we compared LSBMD among participants in arm B between subgroups with no PrEP use (N = 17) and PrEP use (N = 25), defined as TFV‐DP levels <350 fmol/punch and ≥350fmol/punch respectively. Median increase in LSBMD was higher in participants with no PrEP use, +0.03 (IQR 0 to 0.06) compared to those with PrEP use, +0.02 (IQR 0 to 0.03) (*p* = 0.03) (Table 3).

**Table 3:** Comparison of median change in bone mineral density and biochemical markers by adherence and randomization arms between baseline and month 6

	Adherence	N	TDF/FTC once daily + Oskept^®^ twice daily		N	TDF/FTC once daily		*p*‐value**
			Median (IQR)	*p*‐value*		Median (IQR)	*p*‐value*	
LSBMD (g/cm^2^)	Poor	22	0.04 (0 to 0.05)	0.66	17	0.03 (0 to 0.06)	0.03	0.99
	Good	15	0.04 (0 to 0.06)		25	0.02 (0 to 0.03)		0.02
LSBMD Z‐score	Poor	22	0.25 (0.10 to 0.30)	0.19	17	0.10 (0 to 0.60)	0.33	0.89
	Good	15	0.40 (0 to 0.50)		25	0.10 (−0.10 to 0.30)		0.01
Calcium (mg/dL)	Poor	24	0 (−0.4 to 0.1)	0.76	17	−0.2 (−0.5 to 0.3)	0.98	0.90
	Good	14	−0.2 (−0.3 to 0.4)		24	−0.15 (−0.5 to 0.1)		0.82
Phosphorus (mg/dL)	Poor	24	0 (−0.3 to 0.5)	0.78	17	0.3 (−0.2 to 0.7)	0.83	0.30
	Good	14	0 (−0.2 to 0.1)		24	0.3 (0.1 to 0.7)		0.11
25 OHD (ng/mL)	Poor	24	−0.6 (−5.3 to 1.5)	**0.02**	17	−1.5 (−2.3 to 3.0)	0.19	0.56
	Good	14	8.1 (−1.5 to 9.4)		24	−2.05 (−3.6 to 0.6)		**0.02**
iPTH (pg/mL)	Poor	24	7.0(−1.8 to 15.0)	0.09	17	3.8 (−9.3 to 13.3)	0.62	0.54
	Good	14	1.8 (−18.6 to 7.0)		24	7.8 (−5.6 to 21.3)		0.12
ALP (U/L)	Poor	24	−2.0 (−8.0 to 7.0)	0.24	17	−4.0 (−17.0 to 1.0)	0.009	0.13
	Good	14	4.0 (0 to 11.0)		24	4 (−3.5 to 11.5)		0.72

$\begin{array}{cc} \text{No PrEP use},\;\text{’}\text{Poor adherence}\text{’} & =\text{Arm A}:\text{TFV level}<350\;\text{fmol}/\text{punch and}/\text{or}\\ & \;{\text{Oskept}}^{®}\;\text{adherence}\leq 50\%\\ & \;\text{Arm B}:\text{TFV level}<350\;\text{fmol}/\text{punch} \end{array}$

$\begin{array}{cc} \text{PrEP use},\;\text{’}\text{Good adherence}\text{’} & =\text{Arm A}:\text{TFV level}\geq 350\;\text{fmol}/\text{punch and}\\ & \;{\text{Oskept}}^{®}\;\text{adherence}>50\%\\ & \;\text{Arm B}=\text{TFV level}\geq 350\;\text{fmol}/\text{punch} \end{array}$

25 OHD, 25 hydroxyvitaminD; iPTH, intact parathyroid hormone; LSBMD, lumbar spine bone mineral density.

* *p*‐value comparing arms stratified by adherence levels

** *p*‐value comparing median change between arms using Wilcoxon rank‐sum test.

In attempt to look at the physiologic effect of vitamin D and calcium supplementation on BMD, we performed exploratory analysis among the subgroup with PrEP use and good supplementation adherence (N = 15) and arm B who had PrEP use (N = 25). Arm A participants had a median change in LSBMD from baseline of +0.04 (IQR 0 to 0.06) compared to group in arm B without supplementation of +0.02 (IQR 0 to 0.03); *p* = 0.02 (Table 3).

Among participants with baseline vitamin D deficiency, median increase in LSBMD in arm A was higher at +0.05 compared to arm B, +0.03 (*p* = 0.04) (Table 4).

**Table 4:** Comparison of median change from baseline to month 6 between arms by 25 OHD status at baseline

25OHD	N	TDF/FTC once daily + Oskept^®^ twice daily		N	TDF/FTC once daily		*p*‐value**
		Median (IQR)	*p*‐value*		Median (IQR)	*p*‐value*	
LSBMD							
<20	13	0.05 (0 to 0.05)	0.51	17	0.03 (0 to 0.03)	0.23	0.04
>20	25	0.04 (0 to 0.06)		25	0.02 (0 to 0.03)		0.31
LSBMD Z‐score							
<20	13	0.30 (0.20 to 0.50)	0.27	17	0.20 (0 to 0.40)	0.25	0.13
≥20	25	0.20 (0.05 to 0.50)		25	0.10 (−0.10 to 0.20)		0.39
25 OHD (ng/mL)							
<20	13	1.5 (−0.6 to 3.8)	0.27	17	0.5 (−2.4 to 3)	0.02	0.25
≥20	25	−1.5 (−5.3 to 2.4)		25	−2.3 (−4.1 to −0.55)		0.95
iPTH (pg/mL)							
<20	13	7.3 (−5.1 to 12.8)	0.99	17	3.8 (−8.2 to 22.3)	0.79	0.76
≥20	25	4.6 (−1.8 to 14.4)		25	7.8(−5.3 to 18.1)		0.92

25 OHD, 25 hydroxyvitaminD; iPTH, intact parathyroid hormone; LSBMD, lumbar spine bone mineral density.

* *p*‐value comparing arms stratified by 25OHD levels.

** *p*‐value comparing median change between arms using Wilcoxon rank‐sum test.

Subgroup analysis among participants with low LSBMD z‐scores at baseline revealed no differences in median increase in LSBMD, in arm A + 0.05 (IQR 0.04 to 0.05), compared to arm B + 0.05 (IQR 0.03 to 0.05) (*p* = 0.84) (Table S1).

Among 22 TGW who completed six months of follow‐up, the median change in LSBMD in those using FHT (n = 13) was no different, +0.044 (IQR 0.024 to 0.051), in comparison to those not using (n = 9), +0.022 (IQR 0.021 to 0.033) (*p* = 0.10) (Table S2).

### 3.3 Serum vitamin D and iPTH concentrations

Serum 25(OH)D was the same at month 6 in the vitamin D/calcium supplemented group, whereas levels were slightly decreased in the non‐vitamin D/calcium supplemented group (*p* = 0.43) as shown in Table 2. At month 6, 25(OH)D was >20 ng/mL in 63% and 56% of arms A and arm B respectively. However, when analysed by PrEP and Oskept^®^ use, serum 25(OH)D increased from baseline in arm A (+8.1 [IQR − 1.5 to 9.4]), but decreased in arm B (−2.05 [IQR − 3.6 to 0.6]) (Table 3, between groups *p* = 0.02).

Among the 14 participants who had high baseline iPTH, at month 6, eight participants (5 with vitamin D deficiency, 3 with vitamin D insufficiency) still had high iPTH (median 86.2, IQR 80.4 to 123.6).

### 3.4 Safety

There were no differences in any clinical features between the two study arms at baseline and no identified clinical adverse effects of vitamin D and calcium in the supplemented group.

## 4 DISCUSSION

To our knowledge, this is the first study investigating the impact of vitamin D and calcium supplementation on BMD specifically among youth receiving TDF as PrEP. Following the implementation of a PrEP demonstration project under Thailand’s universal health coverage program among MSM and TGW, its use has increased nationwide [21]. Two‐thirds (67.6%) of participants receiving vitamin D/calcium supplementation had ≥3% increase in LSBMD in contrast to 42.9% of those in the control group (*p* = 0.03). Deficits in bone mass accrual during adolescence may result in increased risk for osteoporosis and fractures in later life [39]. Vitamin D and calcium supplementation should be considered in Thai adolescents due to their risk of vitamin D deficiency resulting from the Thai cultural aesthetic preference for pale skin and thus avoidance of sunlight, in addition to typical diets that are insufficient in its dietary intake.

This study was conducted among youth who are in a period of peak bone mass accrual. Young PrEP users in this cohort saw increased BMD measures six months after initiating PrEP. In arm B who did not receive supplementation, we found that those with PrEP use had poorer BMD accrual compared to those with no PrEP use. An adolescent PrEP safety study by Hosek *et al*. [12] observed a 0.23% reduction in spinal BMD z‐scores in youth with a median age of 20.2 years by 24 weeks of follow‐up, also seen in adult PrEP studies with median ages of 20 to 40 years, that reported TDF use to be associated with reduced BMD in HIV negative participants [10, 11, 40]. It must be noted, however, that this study was not designed to specifically look for a relationship between TFV‐DP and BMD and therefore not sufficiently powered to draw reliable conclusions in this aspect. A study on the effect of the contraceptive DMPA on BMD among adolescent girls over a duration of up to two years found that it was associated with −0.33 decline in LSBMD z‐scores [41]. Our study did not see such a reduction in BMD in association with PrEP use or any fractures, but a proportion of study participants did not achieve the expected +3% BMD accrual. This being said, the clinical significance of these findings is dependent on the duration and consistency of PrEP use. At the moment, no data exist on the long‐term BMD outcomes of PrEP use and data from this study provide an important “first look” into the potential trends that could provide some preliminary information on possible outcomes adolescent PrEP providers should be aware of when providing care for longer‐term adolescent PrEP users.

We observed a higher increase in LSBMD at month 6 among participants in arm A with vitamin D and calcium supplementation. However, as vitamin D level results were not immediately available, we did not have the opportunity to supplement vitamin D to those with deficiencies at baseline, and the vitamin D daily supplement provided was in a low‐dose form. As there are currently no published studies to our knowledge on the effect of vitamin D supplementation in PrEP users on BMD, we compared BMD changes of our study to previous studies in HIV‐infected populations, which found vitamin D supplementation reduced BMD reduction on those exposed to TDF containing regimens [28, 32].

One‐third of youth in this cohort had vitamin D deficiency and one‐fifth had low BMD. Although plenty of UVB is available in Thailand, most Thai youth have sedentary lifestyles and avoid sun exposure due to cultural preference for pale skin, contributing to vitamin D deficiency. These observations are in keeping with previous studies that report vitamin D deficiency prevalences of 20% to 30%, suggesting the likely benefit of providing supplementation in adolescent PrEP using populations [24, 42].

In general, vitamin D and calcium supplementation is well‐tolerated, safe, and can be taken over extended periods. However, its dosing was challenging for youth in this study needing self‐administration twice daily in terms of its inconvenience and medication stigma. Vitamin D and calcium supplement adherence in this study was 66%. With recommended vitamin D supplemental doses of 50 000 IU per week [43], lower frequency of administration at higher doses are a potential solution to improve youth supplementation compliance [44, 45] as seen in youth living with HIV where 100% adherence was seen in once monthly vitamin D 50 000 IU dosing which was able to reliably increase 25‐OHD concentrations [19, 32]. In addition, to reduce pill burden, calcium supplementation could be omitted as it is known that no additional benefit is seen on BMD at a higher dose of vitamin D [46]. There is additionally evidence to suggest that if vitamin D levels are low (<30 ng/mL) calcium absorption is impaired [47], suggestive of the relative importance of vitamin D supplementation in comparison to calcium supplementation in BMD maintenance for PrEP users. We observed a non‐significant trend towards increased 25(OH)D levels from baseline to month 6 with vitamin D 400 IU/day, suggesting a possible contribution of insufficient supplementation due to the low dose of supplementation or poor compliance.

We observed that vitamin D and calcium supplementation affected BMD but not serum 25(OH)D, calcium and iPTH levels, as vitamin D is an essential vitamin to maintain PTH levels to stabilize serum calcium [48, 49]. It has been reported that increased vitamin D and/ or calcium intakes have been associated with a positive correlation with BMD despite the normal calcium levels which are controlled by several factors, such as serum albumin and acid‐base status [50]. Many studies demonstrated that BMD is not correlated with 25 OHD levels [51, 52] because serum 25 OHD levels reflect current vitamin D status while BMD denotes bone mineral accrual over a period of time, and is also dependent on other factors such as movement or sun exposure.

The strength of this study is that it provides insights on the effect of daily TDF PrEP to bone accrual among youth and vitamin D and calcium supplementation on BMD of young PrEP users. It has demonstrated that youth generally need supplementation, and strategies are needed to simplify dosing regimens for both vitamin D and calcium to address poor adherence [53], or tenofovir alafenamide, which has a superior bone safety profile compared to TDF needs to be considered [54] to reassure PrEP prescribers and its youth users of its safety on BMD when taking with appropriate supplementation.

This study also had a number of limitations. As its follow‐up period was only six months, we do not have long‐term information as is available from adult population studies that have found bone loss in PrEP use plateaus over time [13, 14]. Mulligan *et al*. [10] observed that the largest decreases typically occur in the first 12 months of TDF/FTC use. Another limitation is the recall and social desirability biases in the three‐day dietary recall data collection method used in the calculation of actual calcium intake. The use of INMUCAL‐Nutrients V4.0 was also mainly based on a western diet and therefore did not particularly align with most of the actual dietary intake of research participants. In the future, it may be appropriate to ensure the adaptation of such measurement tools to the local context prior to their use. Another point is that 21 participants in this study were lost to follow‐up, 13 participants in arm A and eight participants in arm B. Although there were no differences seen between arms, there were higher than anticipated numbers of participants lost to follow‐up. Despite this, there was sufficient power in this study to see a difference between the two study arms. Due to overall low adherence to PrEP, vitamin D and calcium in this study, the overall outcomes of this study may have seen a smaller than expected difference between the study arms. However, when subgroup analysis was performed on those with PrEP use and good vitamin D/calcium adherence, a difference was seen between the groups. This being said, the absence of a placebo control arm in this study ultimately means that apparent benefits seen cannot be fully ascribed to vitamin D and calcium supplementation. A further randomized control trial is needed to clarify this effect. Finally, one‐third of participants had vitamin D deficiency at baseline, which was not corrected until month 6, due to the conduct of retrospective laboratory testing. This, however, reflects real‐life practice where vitamin D measurements are not routinely conducted prior to PrEP initiation.

## 5 CONCLUSIONS

Thai youth who received a daily oral TDF/FTC PrEP regimen had low intake of dietary calcium. The increase in LSBMD over six months among youth who received PrEP who received vitamin D/calcium supplementation was greater than in the non‐supplemented group. Long‐term follow‐up should be considered to explore the long‐term BMD outcomes of young PrEP users. From a PrEP programmatic standpoint, routine supplementation of vitamin D/calcium should be considered among youth taking TDF/FTC once‐daily PrEP, particularly in countries where the majority of youth have inadequate intakes of vitamin D and calcium.

## COMPETING INTERESTS

The authors have no conflicts of interest to declare.

## AUTHORS’ CONTRIBUTIONS

KP was responsible for study design, conduct and writing the initial version of the manuscript. WNS oversaw study participants and supported manuscript writing. ST was a consultant of BMD interpretation. PW coordinated the study and did the data collection. SK did the data analysis. VS was a consultant in the endocrine point of view. TP and SA supervised the overall study design, conduct, data analysis and manuscript writing process. All authors have read and approved the final version of this manuscript.

## Supporting information


**Table S1.** Comparison of median change from baseline to month 6 between arms by BMD z‐score status at baseline
**Table S2.** Comparison of median change from baseline to month 6 among participants using and not using feminizing hormone therapy
