# CORRIGENDUM

**DOI:** 10.1002/jia2.25657

**Published:** 2020-12-18

In the article [1], there were errors in the results section of the abstract and Table 2 on e25624.

1. In the results, the 3^rd^ sentence reads:

**Table 2:** Comparison of bone mineral density and biochemical makers by randomization arm

Parameters	TDF/FTC once daily + Oskept^®^ twice daily (N = 37)			TDF/FTC once daily (N = 42)			*p*‐value**
	Median (IQR)	Change median (IQR)	*p*‐value*	Median (IQR)	Median change (IQR)	*p*‐value*	
LSBMD (g/cm^2^)							
Month 0	0.92 (0.82 to 1.00)	0.04 (0.02 to 0.05)	<0.001	0.95 (0.85 to 1.01)	0.02 (0.01 to 0.03)	<0.001	0.02
Month 6	0.97 (0.84 to 1.03)			0.98 (0.86 to 1.05)			
LSBMD z‐score							
Month 0	−0.9 (−1.7 to −0.4)	0.3 (0 to 0.4)	<0.001	−0.6 (−1.5 to −0.1)	0.1 (−0.1 to 0.3)	0.007	0.25
Month 6	−0.6 (−1.4 to −0.2)			−0.6 (−1.4 to 0.2)			
25OHD, ng/mL							
Month 0	21.3 (18.9 to 26.0)	0.1 (−4.9 to 3.6)	0.92	21.2 (17.2 to 24.9)	−1.7 (−3.1 to 1.5)	0.08	0.43
Month 6	21.5 (18.3 to 25.8)			20.4 (16.0 to 24.4)			
iPTH, pg/mL							
Month 0	38.7 (31.3 to 53.5)	4.9 (−4.2 to 13.5)	0.08	40.8 (30.8 to 57.8)	5.8 (−6.7 to 20.8)	0.07	0.77
Month 6	44.1 (33.1 to 64.6)			45.0 (35.4 to 62.6)			
Calcium, mg/dL							
Month 0	9.9 (9.6 to 10.1)	−0.1 (−0.4 to 0.1)	0.11	9.9 (9.5 to 10.0)	−0.2 (−0.5 to 0.1)	0.11	0.93
Month 6	9.6 (9.4 to 10.0)			9.7 (9.4 to 9.9)			
Phosphorus, mg/dL							
Month 0	3.3 (3.1 to 3.9)	0 (−0.3 to 0.4)	0.57	3.5 (3.0 to 3.7)	0.3 (0 to 0.7)	0.001	0.06
Month 6	3.5 (3.2 to 3.8)			3.7 (3.4 to 3.9)			
ALP, U/L							
Month 0	69 (59 to 87)	−0.5 (−8 to 7)	0.94	79 (66 to 87)	−1 (−7 to 8)	0.62	0.68
Month 6	71 (57 to 88)			76 (65 to 86)			

25 OHD, 25 hydroxyvitamin D; intact parathyroid hormone; iPTH; LSBMD; lumbar spine bone mineral density.

* *p*‐value comparison between months 0 and 6 within arms done with the Wilcoxon signed‐rank test.

** *p*‐value for comparing median difference between arms done with the Wilcoxon rank‐sum test.

The median amount of calcium intake from nutritional three‐day recall was 167 (IQR 94 to 272) mg/day, 39% of participants had vitamin D deficiency, defined as 25(OH)D levels <20 IU/mL.

The sentence should have read:

The median amount of calcium intake from nutritional three‐day recall was 167 (IQR 94 to 272) mg/day, 39% of participants had vitamin D deficiency, defined as 25(OH)D levels <20 ng/mL.

- In the results, the last sentence reads:

There were significantly higher increases in LSBMD among youth with vitamin D deficiency who were supplemented; arm A + 0.05 (0 to 0.05) compared to arm B + 0.03 (−0.1 to 0.03), *p* = 0.04.

The sentence should have read:

There were significantly higher increases in LSBMD among youth with vitamin D deficiency who were supplemented; arm A + 0.05 (0 to 0.05) compared to arm B + 0.03 (0 to 0.03), *p* = 0.04.

- In Table 2, there were numerical errors. The correct Table is shown below:
