# Supplementary material for: Effects of vitamin D and calcium supplementation on bone mineral density among Thai youth using daily HIV pre‐exposure prophylaxis
Source: J Int AIDS Soc. 2020 Oct 11;23(10):e25624. doi: 10.1002/jia2.25624 (PMC7548100; doi:10.1002/jia2.25624)
Supplement: Supplementary file 1 — Table S1. Comparison of median change from baseline to month 6 between arms by BMD z‐score status at baseline Table S2. Comparison of median change from baseline to month 6 among participants using and not using feminizing hormone therapy [file JIA2-23-e25624-s001.docx]

**Supplementary Appendix**

Table S1. Comparison of median change from baseline to month 6 between arms by BMD z-score status at baseline

|  | LSBMD  Z-score | TDF/FTC once daily + Oskept® twice daily | TDF/FTC once daily daily | P-value^*^ |
| --- | --- | --- | --- | --- |
|  |  | Median (IQR) | Median (IQR) |  |
| LSBMD | Z-score ≤ -2 | 0.05 (0.04 to 0.05) | 0.05 (0.03 to 0.05) | 0.84 |
| (g/cm2) | Z-score > -2 | 0.04 (0.01 to 0.05) | 0.02 (0 to 0.03) | **0.04** |
| LSBMD | Z-score ≤ -2 | 0.30 (0.20 to 0.50) | 0.10 (-0.10 to 0.30) | 0.45 |
| Z-score | Z-score > -2 | 0.25 (-0.10 to 0.40) | 0.20 (0.10 to 0.30) | 0.51 |
| Calcium | Z-score ≤ -2 | 0 (-0.40 to 0.40) | 0 (-0.40 to 0.30) | 0.89 |
| (mg/dl) | Z-score > -2 | -0.20 (-0.40 to 0.10) | -0.20 (-0.50 to 0.10) | 0.83 |
| Phosphorus | Z-score ≤ -2 | 0.10 (-0.30 to 0.40) | 0.40 (-0.30 to 0.70) | 0.31 |
| (mg/dl) | Z-score > -2 | 0 (-0.20 to 0.40) | 0.30 (0.10 to 0.70) | 0.10 |
| 25OHD | Z-score ≤ -2 | 0.20 (-3.30 to 4.50) | -3.50 (-3.60 to 3.00) | 0.55 |
| (ng/ml) | Z-score > -2 | -0.60 (-5.30 to 2.40) | -1.65 (-2.85 to 1.35) | 0.57 |
| iPTH | Z-score ≤ -2 | -5.10 (-18.60 to -1.40) | -22.40 (-43.60 to 13.20) | 0.74 |
| (pg/ml) | Z-score > -2 | 7.30 (1.80 to 14.40) | 7.20 (-4.30 to 21.30) | 0.83 |
| ALP | Z-score ≤ -2 | -2.00 (-13.00 to 4.00) | -3.00 (-7.00 to 10.00) | 0.74 |
| (U/L) | Z-score > -2 | 0 (-5.00 to 11.00) | -1.00 (-7.50 to 7.00) | 0.48 |

LSBMD; lumbar spine bone mineral density, 25OHD; 25 hydroxyvitaminD , iPTH; intact parathyroid hormone

^*^P-value comparing median change between arms using the Wilcoxon rank-sum test.

LSBMD z-score calculated using sex- and age-matched references values from Kelly TL, et al. Pediatric BMD reference database for US white children. Bone. 2005:36;S30.

Table S2. Comparison of median change from baseline to month 6 among participants using and not using feminizing hormone therapy

|  | Estrogen exposed (N=13) | Non estrogen exposed (N=9) | P-value^*^ |
| --- | --- | --- | --- |
| Median (IQR) LSBMD change | 0.044 (0.024 to 0.051) | 0.022 (0.021 to 0.033) | 0.10 |
| Median (IQR) LSBMD Z-score change | 0.2 (0 to 0.3) | 0.1 (-0.1 to 0.1) | 0.14 |

LSBMD; lumbar spine bone mineral density

^*^P-value comparing median change between groups using the Wilcoxon rank-sum test.
